# Dopamine release, diffusion and uptake: A computational model for synaptic and volume transmission

**DOI:** 10.1371/journal.pcbi.1008410

**Published:** 2020-11-30

**Authors:** Kathleen Wiencke, Annette Horstmann, David Mathar, Arno Villringer, Jane Neumann

**Affiliations:** 1 IFB Adiposity Diseases, Leipzig University Medical Center, Germany; 2 Department of Neurology, Max Planck Institute for Human Cognitive and Brain Sciences Leipzig, Germany; 3 Department of Psychology and Logopedics, Faculty of Medicine, University of Helsinki; 4 Department of Psychology, Biological Psychology, University of Cologne, Cologne, Germany; 5 Clinic of Cognitive Neurology, University Hospital Leipzig, Germany; 6 Mind & Brain Institute, Berlin School of Mind and Brain, Humboldt-University, Berlin, Germany; 7 Department of Medical Engineering and Biotechnology, University of Applied Sciences, Jena, Germany; École Normale Supérieure, College de France, CNRS, FRANCE

## Abstract

Computational modeling of dopamine transmission is challenged by complex underlying mechanisms. Here we present a new computational model that (I) simultaneously regards release, diffusion and uptake of dopamine, (II) considers multiple terminal release events and (III) comprises both synaptic and volume transmission by incorporating the geometry of the synaptic cleft. We were able to validate our model in that it simulates concentration values comparable to physiological values observed in empirical studies. Further, although synaptic dopamine diffuses into extra-synaptic space, our model reflects a very localized signal occurring on the synaptic level, i.e. synaptic dopamine release is negligibly recognized by neighboring synapses. Moreover, increasing evidence suggests that cognitive performance can be predicted by signal variability of neuroimaging data (e.g. BOLD). Signal variability in target areas of dopaminergic neurons (striatum, cortex) may arise from dopamine concentration variability. On that account we compared spatio-temporal variability in a simulation mimicking normal dopamine transmission in striatum to scenarios of enhanced dopamine release and dopamine uptake inhibition. We found different variability characteristics between the three settings, which may in part account for differences in empirical observations. From a clinical perspective, differences in striatal dopaminergic signaling contribute to differential learning and reward processing, with relevant implications for addictive- and compulsive-like behavior. Specifically, dopaminergic tone is assumed to impact on phasic dopamine and hence on the integration of reward-related signals. However, in humans DA tone is classically assessed using PET, which is an indirect measure of endogenous DA availability and suffers from temporal and spatial resolution issues. We discuss how this can lead to discrepancies with observations from other methods such as microdialysis and show how computational modeling can help to refine our understanding of DA transmission.

## Author summary

The dopaminergic system of the brain is very complex and affects various cognitive domains like memory, learning and motor control. Alterations have been observed e.g. in Parkinson’s or Huntington’s Disease, ADHD, addiction and compulsive disorders, such as pathological gambling and also in obesity. We present a new computational model that allows to simulate the process of dopamine transmission from dopaminergic neurons originated in source brain regions like the VTA to target areas such as the striatum on a synaptic and on a larger, volume-spanning level. The model can further be used for simulations of dopamine related diseases or pharmacological interventions. In general, computational modeling helps to extend our understanding, gained from empirical research, to situations where in vivo measurements are not feasible.

## Introduction

**Fig 1:**
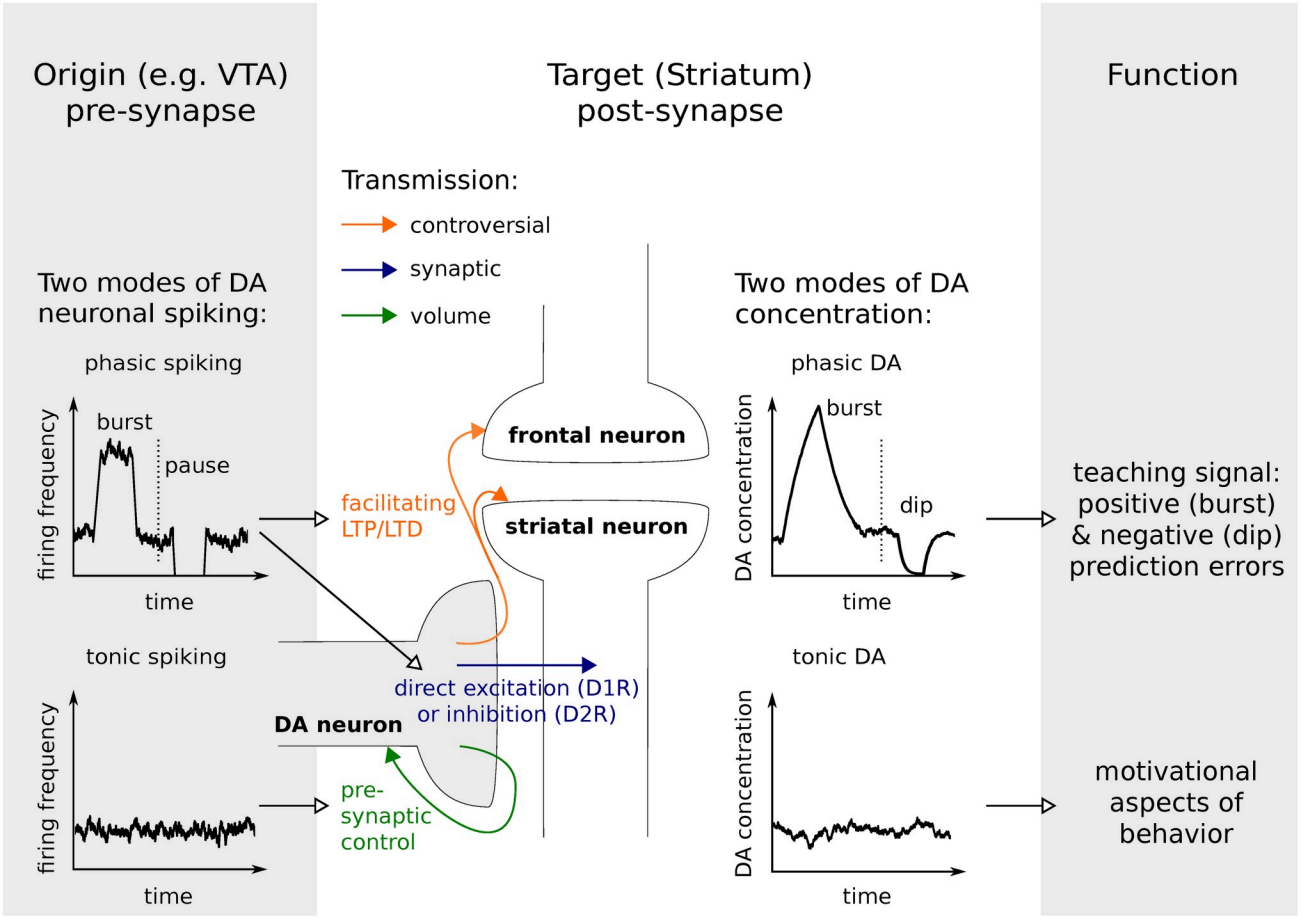
DA spiking patterns and transmission influence cognition and behavior. DA terminal co-localized with a fronto-striatal connection and the two modes of spike-dependent DA release: tonic and phasic spiking. Synaptic spillover facilitates volume transmission. Tonic spiking resulting in tonic DA concentration is primarily associated with volume transmission relevant for pre-synaptic control and, on a functional level, with a motivational drive. Phasic DA acts also pre-synaptically via volume transmission and might be involved in terminating phasic spiking signals. However, phasic spiking, i.e. bursts and pauses, and phasic DA are functionally more relevant for learning. Increased and decreased prevalence of synaptic transmission between DA neuron terminals and specific target post-synaptic neurons contribute to post-synaptic cell excitability. In addition, synaptic spillover following burst firing can increase extra-synaptic DA concentration and modulate fronto-striatal loop connections. Depending on how localized this signal is, the definition of volume transmission may not apply.

The neurotransmitter dopamine (DA) impacts on a variety of cognitive functions (e.g. learning, working memory) and behavioral features (e.g. motivation, affective behavior) that interact in complex ways. While learning demands very precise input-(action-)output-associations, motivation might be thought of as a more general, behavior modulating factor. Two types of dopaminergic signaling, phasic and tonic DA, have been identified in target areas of DA neurons such as striatum or cortex and putatively subserve distinct functions. They are frequently associated with a teaching signal and motivational drive respectively [1, 2] and arise from distinct spiking patterns of midbrain dopaminergic neurons (Fig 1). Tonic DA is grounded in irregular, low frequency firing, whereas phasic signals arise from simultaneous activity in a (sub-)population of neurons. The two characteristics of dopaminergic signaling, tonic and phasic, strongly interact. While prolonged phasic activity can increase the slowly varying levels of DA tone [3], a potentiation of tonic efflux can attenuate phasic signals [4, 5].

**Fig 2:**
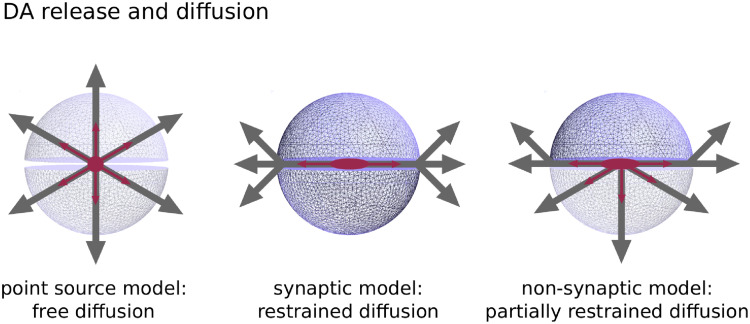
Synaptic transmission. Difference between point source model, synaptic model and non-synaptic model.

DA transmission refers to the process shaping the DA signal. For transmission of DA from dopaminergic neurons to their target areas, synaptic and volume transmission have to be considered differentially. Synaptic transmission describes the precise signaling between two cells. Volume transmission refers to communication between neurons beyond the synaptic cleft (i.e. spillover of DA into extra-synaptic space) and to non-synaptic transmission. The presence of extra-synaptic DA receptors and non-synaptic terminals corroborates the occurrence of volume transmission [6, 7], which has been investigated with different computational models [8–10]. These models commonly rely on a point source model for synaptic terminals, which assumes equal diffusion in all directions, and are thus adequate for non-synaptic, e.g. somatodendritic release (Fig 2). Not considered is the confinement of DA efflux by the geometry of a synapse. However, DA diffusion from synaptic terminals and its impact on DA transmission is still not fully understood and might be crucial to explain apparent discrepancies across empirical findings [11]. It has been proposed that reuptake by DA transporters (DAT) strongly limits diffusion from the synaptic cleft [12]. In turn, DA from the synaptic cleft activates receptors in close proximity to release sites in contrast to volume transmission [13]. In that view, synaptic DA transmission targets precise input-output representations while volume transmission produces a more global signal. Thus, similarly to phasic and tonic signaling, synaptic and volume transmission may impact on learning and motivational aspects of behavior, respectively (Fig 1).

Besides disregarding the precise geometry of a synapse, the aforementioned models rely on a second simplification: the analytical solution solving the partial differential equation for DA concentration is based on the assumption that DA concentration is much smaller than the dissociation constant of DAT, the concentration at which 50% of DATs are occupied. However, this holds only for relatively small values of DA concentration like normal tonic DA levels, but not in close proximity to terminals after DA release or in scenarios of strong tonic DA increase, for example after amphetamine or cocaine administration. In particular, under this assumption Michaelis-Menten uptake becomes linear and uptake will be overestimated, i.e. DA concentration will be underestimated.

Here we present a new DA transmission model, a simulation tool for multiple terminal DA release, diffusion and uptake that incorporates the specific geometry of synapses (Fig 2A right). Furthermore, while the model is based on the same partial differential equation as previous models, it does not restrict on certain concentration values as it relies on a full numerical approach with the method of finite elements, in contrast to analytical approaches.

The paper is structured as follows. First, in section *’Synaptic transmission’* we focus on our small-scale model encompassing a single synapse and its near-synaptic space. We examine how strongly DAT uptake actually acts near a synaptic cleft. This has been a matter of debate so far [14, 15], whereby Floresco and colleagues urge to consider the synaptic and extra-synaptic compartment of the DA system differentially. However, from previous point source models of DA transmission one can not distinguish between these two compartments. We model the potential effect on DA-type2-receptors (D2R), because this allows to relate our simulations to empirical findings using methods based on DA action at these receptors such as [11C]raclopride positron emission tomography (PET). In earlier models Michaelis-Menten kinetics for D2R have been used to quantify D2R occupancy. However, these models assume steady state concentrations. More recently, a dynamic receptor binding model has been proposed [16], incorporating on- and off-kinetics of the receptors. In their paper, the authors use parameters from the literature that suggest slow kinetics. However, they also mention the possibility of faster kinetics implied by recent findings using optical methods. Our new computational model enables us to discuss both, slow and fast binding.

Second, in section *’Spatio-temporal variability of tonic DA concentration in extra-cellular space’* we examine simulations of our large-scale model, which contains ∼ 13000 synaptic and non-synaptic terminals. We compare simulations of increased DA release and of uptake inhibition to our default simulation and focus on temporal and spatial variability in extra-synaptic space. In addition, we test a hypothesis postulated by Laruelle [11] that bridges apparent discrepancies between observations using PET versus microdialysis. This and similar discrepancies across studies may be traced back to resolution issues of different empirical methods. In this context, we will discuss the two extra-cellular compartments of DA transmission: intra-synaptic and extra-synaptic DA. A full mathematical account of our model and a complete list of model parameters are presented in Section *’Materials and methods’* and in in S1 Table, respectively.

## Results and discussion

### Synaptic transmission

Synaptic DA transmission may be of particular importance for understanding the role of DA where striatal DA synapses occur interrelated with synapses from limbic and cortical inputs. Here, DA is of crucial importance for long-term modulation of connections [17–19] and ultimately for cognitive functions like reward processing and learning. Empirical studies suggest that the synaptic micro-environment needs special consideration. Laruelle [11] discussed the possibility that if a challenge for benzamide-D2R binding primarily occurs within the synaptic space, this would partly explain discrepancies across pharmacological agents (nicotine vs. amphetamine) in their ability to affect DA microdialysis and benzamide binding potential measurements. Concerning also the synaptic level, Beckstead et al. [13] suggested a special role for synaptic transmission in directly regulating post-synaptic cell excitability. From cell recordings in mice midbrain slices the authors concluded and summarized that “synaptic DA transmission does not depend on volume transmission” and that “uptake is a critical factor controlling shape and duration of inhibitory post-synaptic currents” [13]. Similar results were recently reported in striatal interneurons and medium spiny neurons (MSNs) using optical methods [20, 21].

According to the suggestion that the synaptic compartment has to be distinguished from the extra-synaptic compartment, in our analysis of synaptic transmission we particularly focused on the hypotheses that (1) DA release is highly localized to the synapses and that (2) DA uptake strongly contributes to this tight localization. This has been a matter of special debate [14, 15] and links to the models of ‘private’ vs. ‘social’ synapses by Sulzer and Pothos [22]. In a ‘private’ synapse molecules of a single release event are restricted to the peri-synaptic area, in contrast to ‘social’ synapses facilitating volume transmission.

The hypotheses were in part addressed with previous computational models. Garris and colleagues [23], for example, considered the geometry of the synaptic cleft but focused on the process of diffusion. The authors aimed at quantifying the maximum possible efflux from the synaptic cleft, and thus disregarded the process of DA uptake. By comparing the fast relaxation time of the DA concentration gradient after release with the half-life of DA uptake, they conclude that diffusion rather than uptake is the dominating process here. Nicholson [24] investigated the dynamics of DA after release from a point source (the tip of a microelectrode) using a model integrating diffusion and uptake simultaneously. He demonstrated solutions for the respective partial differential equation assuming linear uptake and a baseline DA concentration equal to zero. To investigate a single terminal release event Cragg and Rice [8, 9] used the same point source model. They conclude that the predominant mode of DA signaling is volume transmission. In contrast, we will argue for a potential role for synaptic terminal release in addition to volume transmission. In the following, we will discuss the influence of the synapse geometry, non-zero baseline concentration, multiple release from a single terminal, as well as homogeneous and non-homogeneous Michaelis-Menten uptake. Finally, we discuss models of receptor occupancy, i.e. equilibrium binding [8, 9] as well as slow and fast dynamic binding [16, 25, 26].

#### A single full content release event

**Fig 3:**
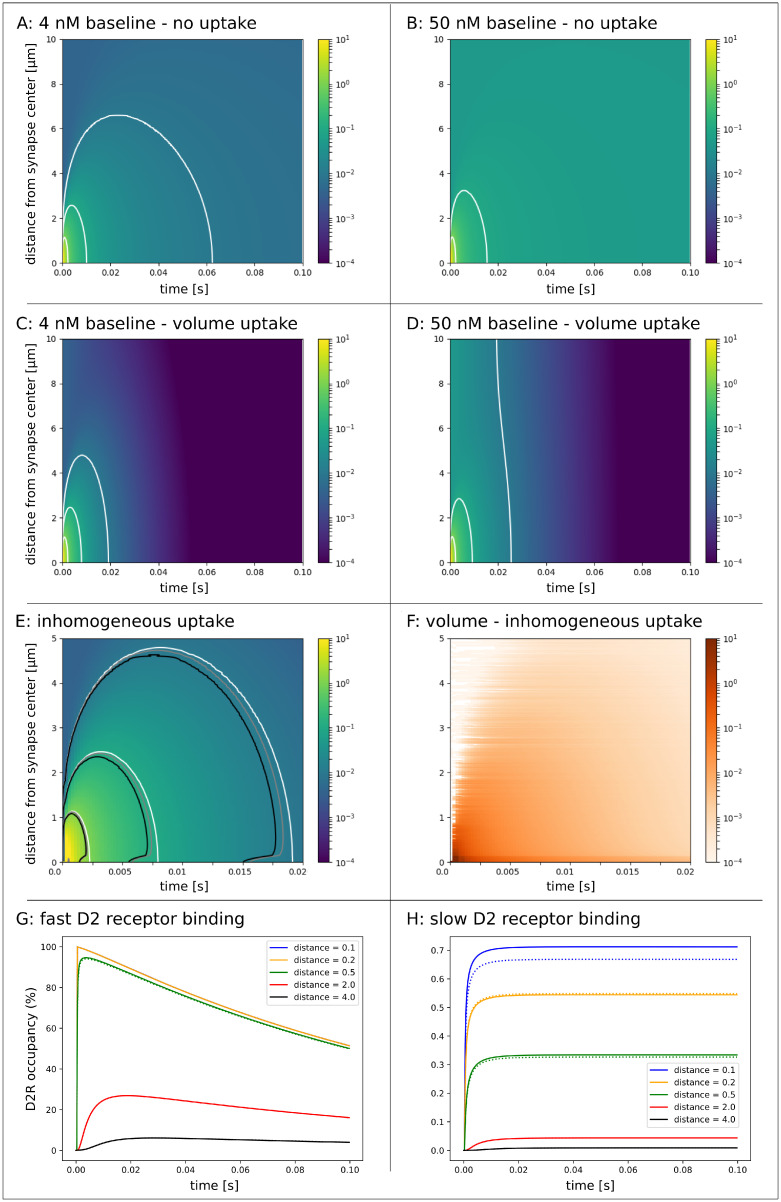
Single DA release event from synaptic terminal. For all plots the initial baseline concentration was 4*nM* except in B & D where the inital baseline concentration was 50*nM*. **A-D:** Release of 3000 DA molecules from synaptic terminal. White isolines indicate the concentration of 10, 100 and 1000*nM*. All color scales represent concentration in μM. DA quickly escapes the synaptic cleft in scenarios without uptake (A & B) and also with volume (homogeneous) uptake (C & D). **E:** Detail of plot C with additional isolines of 10, 100 and 1000*nM* for simulations with a combination of volume and surface uptake (inhomogeneous uptake). White isolines refer to the simulation with volume uptake only. Gray and black isolines refer to the simulation where surface uptake was five and one-hundred times elevated, resp. Neither homogeneous uptake nor uptake strongly pronounced at the pre-synaptic terminal can prevent synaptic spillover. **F:** Concentration difference between simulations of homogeneous (white) and inhomogeneous uptake with five (gray) and one-hundred (black) times elevated uptake at the pre-synaptic terminal. **G:** Fast D2 receptor binding at different distances from release site. Comparable receptor binding occurs at distances up to 0.5μm. The blue and yellow graph are equivalent. D2R binding at half the distance of neighboring terminals (2.0μm) is still profound but negligible at 4.0*μM*. Differences between a synaptic (solid line) and a non-synaptic terminal (dashed line) could not be confirmed. **H:** Slow D2 receptor binding at different distances from release site for a synaptic (solid line) and a non-synaptic terminal (dashed line). Elevated receptor binding occurs at a distance of 0.1μm, (i.e. inside the synapse for the synaptic terminal with radius = 0.15μm), but not at distances beyond 0.2μm. D2R binding beyond half the distance of neighboring terminals (2.0μm) is small and negligible at 4.0μm.

First, we simulated a single terminal release event with and without homogeneous Michaelis-Menten uptake kinetics, where the full vesicle content of 3000 molecules was released from the center of a synaptic terminal. Initial baseline concentration was chosen to be at the extreme ends of the range of empirical measures (4*nM* and 50*nM*). DA quickly escapes the synaptic cleft, which can not be prevented by homogeneous uptake as illustrated in Fig 3A–3D. White isolines indicate DA concentration of 1000, 100 and 10*nM*. DA concentration higher than 1000, 100 and 10*nM* lasts for 2, 8 and 19*ms*, respectively, in the 4*nM* baseline scenario with volume uptake and for 2, 9 and 25*ms*, resp., in the 50*nM* baseline scenario with volume uptake. These different values between the two baseline scenarios arise because it takes more time in the 50*nM* scenario to decrease concentration in the entire volume. The clearing of the synapse after DA release does not actually depend on baseline concentration values. The concentration gradient is extremely steep such that in scenarios of baseline concentrations between 0.001 and 500*nM* more than 97% of the released molecules have left the synaptic cleft within 0.25*ms*.

Moreover, isolines in the plots and hence the DA concentration patterns differ between scenarios of no vs. homogeneous uptake. There is no 10*nM* isoline in the scenario of 50*nM* without uptake. However, this isoline appears in the scenario with uptake. Thus, DATs contribute substantially to the overall DA flux and facilitate the balance towards DA release. Since, in striatal regions, DAT is localized on axons and axonal terminals near and distant from synaptic junctions [27, 28], volume averaged uptake is a simplified model, that does not reflect the true localization and dynamics of uptake. The question remains, whether increased DAT activity at the synaptic level can prevent synaptic spillover of DA. Thus, we ran a further simulation of this micro-environment and tested the effect of enhanced uptake on the cell membrane surface of the pre-synaptic terminal. In comparison to volume uptake, this surface uptake acts very localized and requires a different set of model parameters. Values for those parameters were chosen to promote very strong surface uptake (see section ‘Materials and methods’). Nonetheless the effect is rather small Fig 3E & 3F. Even though there is a DA concentration difference up to few *μM* between homogeneous uptake (volume uptake only) and inhomogeneous uptake (surface and volume uptake), the isolines do not shift substantially. We conclude that even strong DAT activity near the synaptic junction can not prevent DA spillover, hence volume transmission plays an important role in DA signaling. However, in line with our second hypothesis, uptake promotes the ‘privacy’ of a synapse. Whether or not there exists cross-talk between neighboring synapses will strongly depend on how we define this inter-synaptic communication in the model.

Cragg and Rice strongly emphasize the role of volume transmission in DA signaling, because the average distance to neighboring synapses, that was previously estimated to be ∼ 4μm [23], is much smaller than the distance at which concentration equals 10*nM* in their model (the ‘outer’ isolines in Fig 3A–3F). Note that in their model they released three times the number of molecules from our model. We adhere to this number in line with other previous models and our simulations of multiple terminal release as discussed in the next section *’Spatio-temporal variability of tonic DA concentration in extra-cellular space’*. All remaining parameters for diffusion, uptake and release are identical (S1 Table). 10*nM* was chosen as the critical value, because it corresponds to the equilibrium constant for D2Rs. In this case, half the D2Rs would be occupied at a steady state concentration of 10*nM*. However, two aspects may be discussed here. First, the model by Rice and Cragg assumed zero baseline concentration. This is inadequate in light of empirical values for tonic DA levels, which can by far exceed the equilibrium constant (up to 50*nM* for rodents and up to 72*nM* for humans, S3 Table). Second, the DA signal is highly transient, which violates the equilibrium assumption for D2R binding. A different opinion is provided by the group around Williams, Beckstead and Ford [13, 29, 30]. Their reasoning is also based on DA acting at D2Rs, but from an empirical perspective that accounts for the dynamic nature of DA release. The authors argue for a post-synaptic mechanism independent of diffusion, in contrast to the principles of volume transmission. Despite the high affinity of D2Rs for DA, a much higher concentration than 10*nM* is required to produce the activation of receptors that would mimic an electrically evoked D2R mediated response in midbrain DA neurons. Transferring this idea to striatum, from an experimental point of view the 10*nM* isoline might not be the best indicator where synaptic spillover of DA affects cell properties. Especially since this value is within the range of baseline concentrations. In our simulations the ‘middle’ isoline of 100*nM* DA concentration, which is less sensitive to baseline concentration, remains below the average distance to neighboring synapses in all scenarios even without uptake. Our simulation with 4*nM* (50*nM*) baseline concentration and homogeneous Michaelis-Menten kinetics yielded a distance of 2.3*μm* (2.8*μm*). The 1000*nM* isoline appears at a distance of 1.14*μm* (1.15*μm*). The next question is how the concentration time course looks like, if a single synapse releases DA successively within a short time interval. After examining multiple release events, we will model and discuss receptor binding in more detail and pick up on this discussion.

#### Multiple releases event from a single terminal

**Fig 4:**
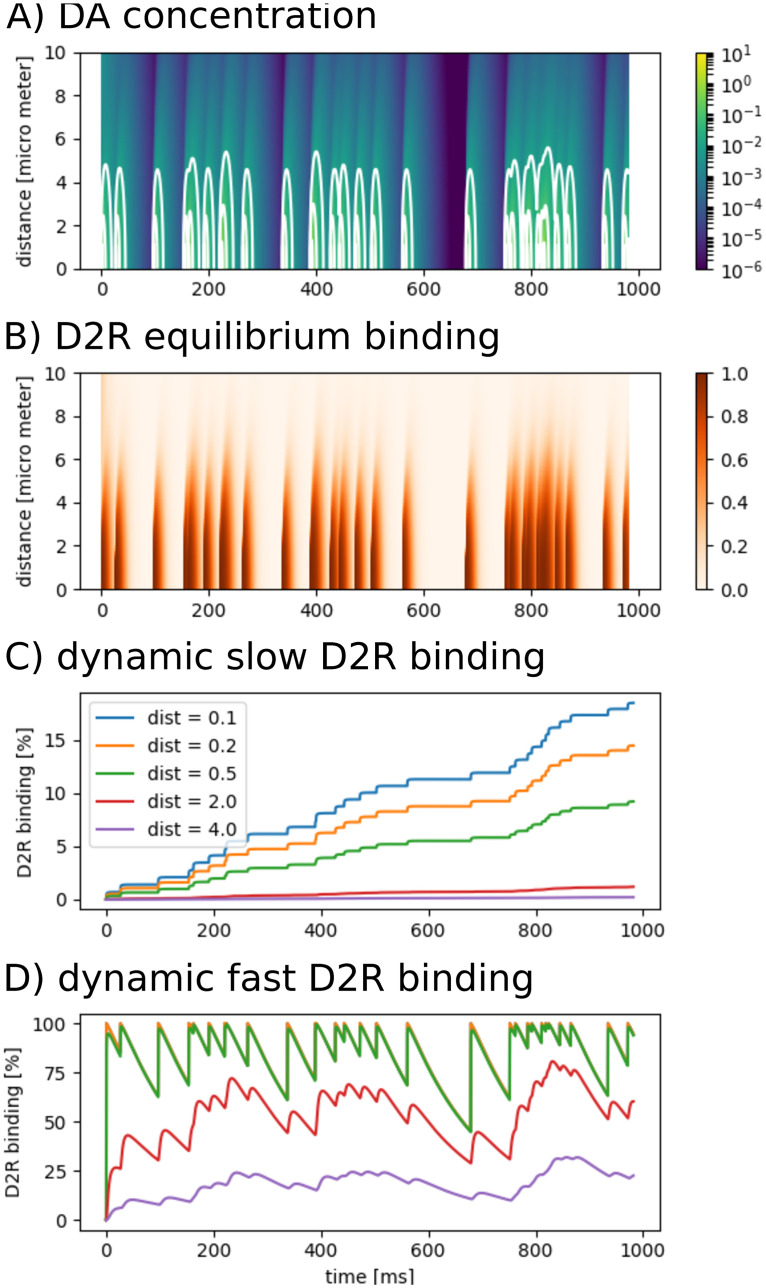
Multiple DA release events from synaptic terminal. **A:** Simulated concentration [μM] with 3000 molecules released per event. **B:** Associated equilibrium receptor binding (Michaelis-Menten kinetics). **C:** Associated slow receptor binding. Percentage of binding increases with each release event inside and close to release site. At 2*μm* distance from the synapse center increments become insignificantly small. **D:** Associated fast receptor binding. Again, at 2*μm* distance from the synapse center receptor binding becomes insignificantly low.

Next, we modeled a single synaptic terminal releasing DA at a frequency of 30*Hz* (Poisson process), which could be considered a burst mode. However, the model does not capture DA input from other terminals that would release DA synchronously in such case. Initial baseline concentration was 4*nM*. An entire vesicle content of 3000 molecules was released each time. Notably, a release probability of approx. 6% or partial content release ranging between 0.1 − 21% has been suggested [10, 31]. Thus our simulation presumably exaggerates realistic DA release. However, between release events DA concentration falls below 10*nM* (Fig 4A). The 10*nM* isoline embraces multiple spike events only if they occur within short intervals (<17*ms*). Hence without the pooling effect of multiple synapses, in the peri-synaptic area of a single terminal DA concentration ≥10*nM* will not endure with release frequencies higher than tonic firing modes. These range between 0.5 and 8*Hz* [32]. Similarly, considering a fraction of release or partial content release such high concentrations do not endure during burst firing, i.e. ∼15 − 50*Hz* in rodents and up to 100*Hz* in primates [32].

#### Dynamic receptor binding

As stated earlier, the debate about the privacy of a synapse may be based on the ability of synaptic DA to bind to receptors outside the synaptic junction. Directly translating concentration values to dopamine receptor binding using Michaelis-Menten kinetics [8, 9] does not take into account the on- and off- binding kinetics when concentration is dynamic. In this case, concentration is simply transformed onto the [0, 1]-interval, reflecting concentration dynamics, but not receptor dynamics (Fig 4B). For transient DA release on the synaptic level we implemented dynamic receptor kinetics as proposed in [16]. Slow binding is illustrated in Fig 4C. In this plot, we assigned colors to different distances from the synapse center. While binding increases stepwise with each terminal release event at small distances (blue, orange, green), this stepwise increase is almost not visible at distances further than 2*μm* (red & purple), which is half the distance to neighboring synapses. With this result we again argue for the privacy of a single terminal. In this scenario of slow binding and 0% initial receptor occupancy, after 1000*ms* 18.5% of the receptors are occupied inside the synapse. Over a long period of time occupancy will reach an asymptote, that will characterize the average DA release-uptake balance of the terminal over time. Changes in firing patterns and consequently in striatal DA release are only detectable over time scales of several seconds to minutes [16].

Recent studies using genetically encoded sensors [25, 26] suggest faster receptor binding. In contrast to the monotonic increase in case of slow receptor binding, in this scenario receptor occupancy is much more dynamic and fluctuates between 44.7% and 100% in synaptic and peri-synaptic space. Also at a distance further than 2*μm* these fluctuations are prominently pronounced ranging between 28.8% and 80.7% (Fig 4E), but not so much at the average distance of neighboring synapses, where D2R occupancy is between 10.1% and 31.8%. With fast binding kinetics a single release event is capable to increase D2R binding by maximum 5.7% (Fig 3G).

#### Synaptic vs. volume transmission

We conclude, DA leaves the synaptic cleft due to high gradients between extra- and intra-synaptic concentration after terminal release, which can not be prevented by DAT even if strongly pronounced at the pre-synaptic terminal. Thus diffusion is the predominant process within in the first milliseconds. However, for the subsequent shaping of the concentration pattern DATs contribute to the privacy of the synapse. In summary, we found with respect to slow binding kinetics that synaptic DA is highly localized to the synapse. The model of fast receptor kinetics shows some effect on D2R binding at the average distance of neighboring synapses. However, with DA contribution from multiple synapses, that are missing here, is remains questionable if synapses actually detect neighboring signals. Thus cross-talk between synapses is limited in the sense that a synapse can not actually eavesdrop on a neighboring synapse. This does not exclude that synapses can detect concerted activity, i.e. the average population DA release in their neighborhood, such as changes in tonic activity as well as changes from tonic to burst modes. Thus both, synaptic and volume transmission may contribute to complex DA signaling.

As we previously touched upon, from an experimental point of view data from midbrain neurons suggest locally induced D2 autoreceptor mediated currents, so-called inhibitory postsynaptic currents (IPSCs). Two scenarios of DA application in midbrain could induce an IPSC comparable to electrical stimulation. Either a DA concentration of 10*μM* for at least 25*ms* or an instantaneous DA concentration of 100*μM*. While this was previously interpreted as a post-synaptic response, Rice and Patel [33] argue that these midbrain DA neurons are autoregulated primarily by their own DA release, rather than via synaptic DA release. Importantly, IPSC have been studied in striatal MSNs [21] and interneurons [20]. Both studies conclude a localized and discrete action of DA on these neurons.

Directly arguing with the empirical data derived from midbrain neurons, a prolonged concentration of >10*μM* that is necessary to mimic the electrically evoked IPSC, does not occur in our simulation of a single striatal synapse. It may be realized via volume transmission through the pooling effect of DA release from multiple terminals (e.g. during burst activity). An instantaneous DA concentration of >100*μM* occurs in our simulation only inside the synapse. Consequently, we conclude that DA acts highly localized to the terminal. However, this is not necessarily restricted to action inside the synapse, but at least to the peri-synaptic area (Fig 4C & 4D), particularly in view of the fact that most receptors are located extra-synaptically. In addition, we designed a non-synaptic terminal and compared DA release between both terminal types (Fig 3G & 3H). Our results show that fast DA spillover from the synapse is not very different from non-synaptic DA release and the discussion about synaptic terminals above apply similarly to non-synaptic terminals. The main difference is the high concentration that occurs briefly within the synapse. The figure illustrates the D2R binding difference between synaptic and non-synaptic terminal DA release (solid versus dotted lines). This difference is apparent only directly next to the release site, i.e. inside the synapse.

### Spatio-temporal variability of tonic DA concentration in extra-cellular space

As a physiological measure, DA tone refers to the endogenous DA concentration in the brain in the absence of neuronal bursts, spatio-temporally averaged according to the resolution of the empirical measurement method. Alteration of DA tone has attracted much attention as it may be crucial for cognitive impairments in neurological disease and psychiatric disorders, including Parkinson’s and Huntington’s Disease [34], addiction [35, 36], pathological gambling [37, 38] and binge eating disorder [38]. Different empirical methods aim at quantifying DA tone, either directly, but invasively (e.g. microdialysis [12, 39–41]) or indirectly (e.g. PET [11, 42–45]).

DA transmission describes the complex interplay between DA release, diffusion and uptake. The resulting DA tone is determined by the sensitive balance between DA release and uptake. Inside the cell DA is stored in vesicles which fuse with the membrane when the neuron spikes (exocytosis), thereby releasing DA into the extra-cellular space. DA release relies on multiple factors. These include DA synthesis and recycling of DA molecules into vesicles (both of which impact on the amount of DA in vesicles), fraction and probability of vesicles content release per spike event, and the frequency at which tonic spiking occurs. DA uptake on the other side of the balance is also influenced by several factors such as DAT localization, DAT trafficking or the DAT recycling rate. Mathematically, these factors are expressed by the Michaelis-Menten uptake kinetics, which in turn relies on two parameters, the maximum uptake rate *V*_*max*_ and the so-called dissociation constant *K*_*m*_ which indicates the DA concentration at which 50% of DATs are occupied.

Extra-synaptic DA concentration is highly heterogeneous throughout space and time. To understand the dynamics of dopaminergic signaling and potential consequences, not only the mean DA concentration but also its variability might be of importance. With our model we can simulate arbitrarily small volumes and high temporal resolution, although at the cost of computational effort, and examine DA distribution throughout the simulation space. This allows to bridge apparent discrepancies from empirical observation across methods, that may arise from resolution problems. In the following, we first examine a default simulation, meant to reflect healthy DA transmission. Second, we focus on temporal variability in extra-synaptic space and show how it differs between a simulation of uptake inhibition, increased DA release and our default simulation. Importantly, the uptake inhibition simulation reaches a concentration level that violates the assumption to be much smaller (<< *K*_*m*_) or much higher (>> *K*_*m*_) than the dissociation constant of DAT (*K*_*m*_ = 210), thus impeding the application of an analytical solution to the differential equation (see ‘Materials and methods’). Third, we discuss why both, temporal and spatial variability, have to be taken into account when assessing receptor occupancy as a function of DA concentration.

Finally, we want to test a hypothesis based on imaging pharmacological interventions using PET, postulated in Laruelle 2000: for agents that enhance DA concentration by inhibiting uptake (e.g. amphetamine), the competition between endogenous DA and radioligand occurs prominently at non-synaptic D2Rs. On the other hand, following the administration of drugs that stimulate DA release (e.g. nicotine) the competition is more pronounced at synaptic D2Rs. Laruelle references studies investigating simultaneous PET and microdialysis measurements, which show similar reductions in raclopride BP between administration of uptake inhibitors and DA release stimulants. However, the associated increases in extracellular DA was much larger following uptake inhibition than following elevated DA release. These results can be expected with a predominant intra-synaptic location of the challenge following increased DA release and microdialysis measuring extra-synaptic DA only, while PET measures both, extra- and intra-synaptic D2R challenges. For the following analysis, we ran simulations in three different parameter settings: (1) default DA, (2) direct DA enhancer and (3) indirect DA enhancer.

#### Default setting

First, as a proof of concept for our model, we simulated DA transmission under default parameter setting. This default setting mimics healthy DA transmission to the striatum, i.e. where DA tone is determined by an undisturbed balance of all subprocesses. To simulate DA transmission to cortical areas an adjustment to various parameters has to be considered. Cortical areas receive much less DA projections compared to striatal areas resulting in much lower DA concentration levels in prefrontal cortex [46].

**Fig 5:**
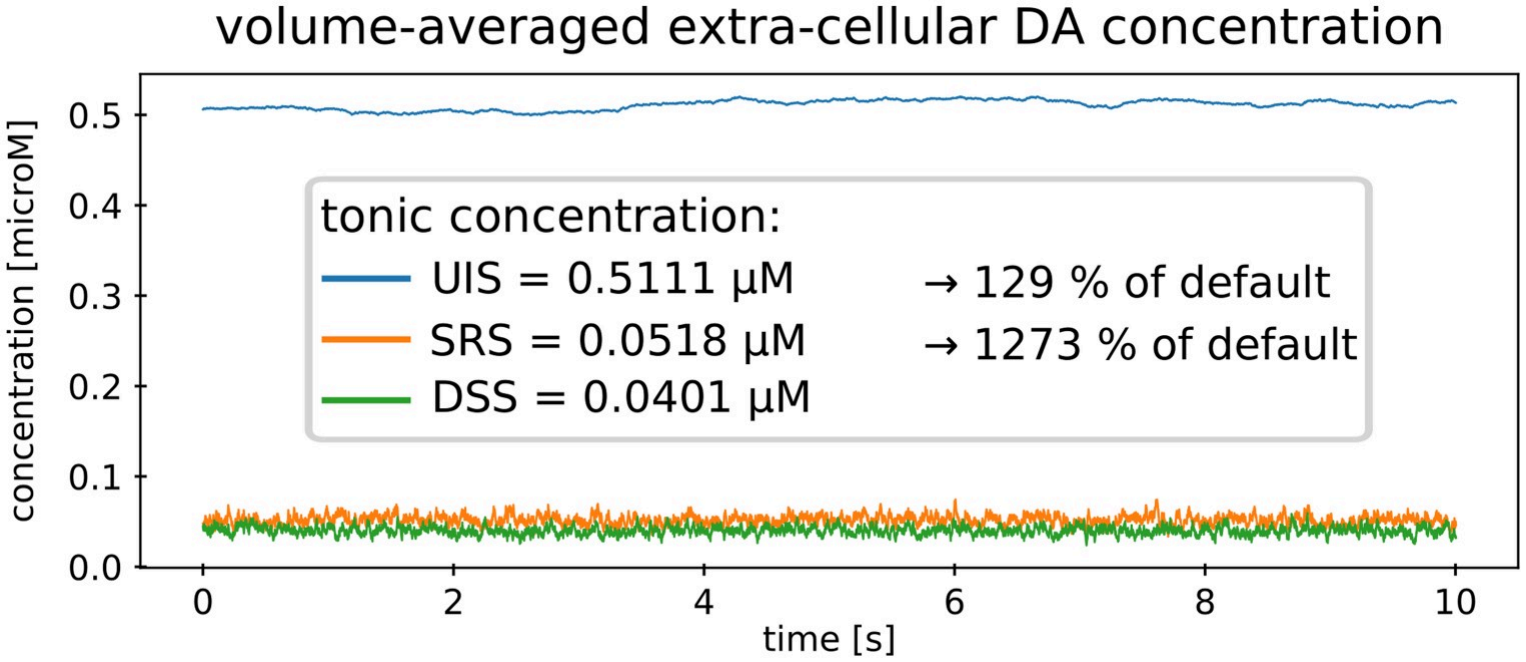
Extra-synaptic DA. Volume-averaged DA concentration for the three different simulation settings. Fast oscillations occur in the DSS and SRS, while low frequency oscillations occur in the UIS. DSS: default setting simulation, SRS: stimulated release simulation, UIS: uptake inhibition simulation.

For reasons of comparability, we choose our default parameter setting in accordance with a previous model by Dreyer and colleagues [10]. Additionally we implemented parameters needed for synaptic specificity (S1 Table). Dreyer’s model resulted in a slightly lower DA tone of ∼ 37*nM*, compared to our model, where the average extra-synaptic concentration is ∼ 40*nM* (Fig 5). The discrepancy between our and Dreyer’s model results can be explained by the approach to solve the partial differential equation describing diffusion and uptake. Dreyer and colleagues used a mixed strategy. Like in our approach, they solved their large-scale model numerically, but in this large-scale model the authors integrated an analytical solution for individual terminal release as it was suggested in [8]. Importantly, this analytical solution holds only for DA concentrations much smaller than the dissociation constant for uptake [24], which is not the case in direct vicinity of terminals. The analytical solution was achieved by assuming linear uptake, where in fact uptake converges to an upper limit due to saturation (see ‘Materials and methods’). Consequently, this assumption overestimates DA uptake for high concentration, i.e. around a terminal after DA release. In contrast, we implemented an overall numerical solution that does not violate the saturation effect of DATs.

DA tone resulting from our default simulation setting is well in the range of empirical measures. Studies with rats report values in striatal regions between 4 − 50*nM* [39–41, 47]. PET or SPECT studies derived human in vivo steady-state DA levels ranging between 23 and 72*nM* (S3 Table). Using the range of empirical measurements for the tonic firing frequency of 2 − 5*Hz* [48], we simulated DA tone and obtained basal DA levels between approx. 20 and 50nM.

In our model several specific implementations have to be discussed. First, fixed parameters in our healthy condition model have been taken from previous models and are not consistently from the same species. It is particularly difficult to find values for all required parameters in humans in order to realistically simulate human endogenous DA levels. Moreover, often parameter values can not be precisely determined from the literature. For example, reported empirical values for the two parameters describing Michaelis-Menten uptake kinetics in the striatum differ widely across and within species (S2 Table). These differences might arise in part from methodological differences in measurements, but could also reflect individual differences and even heterogeneity within an individual’s striatum [49, 50]. Second, for a single terminal there is evidence that not every spike is associated with an event of exocytosis and for such events the vesicle content might not be released entirely. This was investigated in detail by Rooney and Wallace [31], but based on their computational model, the authors could not conclude which mechanism is actually functional in the brain. However, assuming partial content release at all terminals ranging between 0.1 − 21% they were able to keep a reasonable tonic DA level constant. With our model we adhere to Dreyer and colleagues and implemented full content release at a fraction (default = 6%) of terminals. Finally, in the living being vesicle content is not released at an instance as in our model [48]

#### Stimulation of DA release and uptake inhibition

To simulate the presence of an agent stimulating DA release, we increased the release probability from 6% to 7.8%. This elevates DA tone to 129% of the DSS, similar to a representative example mentioned by Laruelle. Here, rodents were administered a high dose of nicotine (5*mg*/*kg*). He compared the results with a study of amphetamine administration (0.4*mg*/*kg*) in rhesus monkeys which elicits a 1365% increase over baseline in extra-synaptic DA as measured with microdialysis. For the UIS we set the maximum uptake rate *V*_*max*_ to 80% of the default and *K*_*m*_ to 24*μM*, as it has been observed after amphetamine superfusion of mice striatal slices using cyclic voltammetry [51]. In addition, we divided the number of molecules being released during an event of exocytosis by 8.5 to obtain approximately the above mentioned increase in DA tone. A reduced number of molecules is a consequence of less DA recycled into the cell and D2R auto-inhibition of stimulation-dependent DA release. Note, however, that we do not simulate the above mentioned interventions themselves. These pharmacological manipulations are much more complex. For example, amphetamine does not only block but also reverse transporters. Hence for a realistic simulation of amphetamine administration stimulation-independent retrograde release via DAT has to be considered. Rather, we are interested in the DAT blocking effect on extra- and intra-synaptic DA concentration compared to promoted DA release and consider simplified models of uptake inhibition and enhanced DA release.

#### Temporal variability

We first consider temporal fluctuation of the volume-averaged extra-synaptic DA concentration, by comparing default setting simulation (DSS) with simulations of uptake inhibition (UIS) and stimulated release (SRS). In Fig 5 extra-synaptic DA for all three simulations are displayed. DSS, SRS and UIS yielded a tonic DA level of ∼40*nM*, ∼52*nM* and ∼511*nM*, respectively. Importantly, in the UIS, DA concentration values are larger than the dissociation constant *K*_*m*_ and DA uptake approximates its maximum (see ‘Materials and methods’).

With respect to temporal variability, we obtained the following results. Firstly, under default conditions and in the SRS, DA tone leveled very quickly compared to the UIS after initialization with high and low concentration values. This indicates an effective balance between release and uptake, capable of reinstating extra-synaptic concentration for example after the occurrence of a burst or pause of neuronal activity. In contrast, DA tone of the UIS needed considerably more time to settle. Secondly, in the default setting and in the SRS extra-synaptic DA concentration oscillated fast around the mean (i.e. DA tone), while in the UIS slower and larger fluctuations appeared.

We examined these fluctuations by fitting an Ornstein-Uhlenbeck process (OUP) to the simulated time series. OUPs are used to describe mean reverting processes and contain three parameters: (1) the mean reversion *level* or balance level (in our application DA tone), (2) the mean reversion *rate*, which measures the strength of attraction towards the balance level (in our application the capability to reinstate DA tone) and (3) the *volatility* parameter describing the size of random jumps (Poisson-distributed spiking). The process was fit to the simulated time series using maximum likelihood estimation. After the fitting procedure, the balance levels of the OUPs corresponded to the means of the extra-synaptic concentration, i.e. ∼ 39.7*nM* for default, ∼ 52.2*nM* for SRS and ∼510.3*nM* for UIS. We further obtained best estimates for the rate and volatility parameter, however, depending on initialization of the optimization algorithm we found multiple local maxima. In total we ran the fitting procedure with nine different initializations. All results and a more detailed discussion of the locally best estimates is provided in S4 Table. In the following we will only report the essential outcomes.

First, we will compare default simulation and stimulated release simulation. We found a difference between the rate parameters, which was larger for the SRS than for the DSS and comparable volatility parameters, being just slightly higher for the SRS. The two simulations differ by an enhanced release frequency in the SRS, while the number of DA molecules is the same for every release event in both simulations. Hence the size of the random jumps, which we associate with the volatility parameter is comparable, while elevated DA release in the SRS requires faster reinstatement (i.e. a higher rate parameter).

For the UIS we found that both, the rate and volatility parameter were lower compared to default and SRS. This is not surprising, given that we reduced the amount of DA molecules being released in the UIS, which should in turn result in smaller random jumps. Moreover, the lower rate parameter for UIS signifies that reinstatement is impaired by lower DAT activity.

In addition, the variance of an OUP is given by the ratio *volatility*^2^/(2 * *rate*). From a mathematical point of view, this ratio is the variance of the stationary distribution of the stochastic process, the OUP. Specifically, it constitutes a relationship between random tonic DA release and the capacity of the system to reinstate stationarity, thus it is the variance of the concentration time course, when the concentration level is around the mean (tonic) DA level, i.e. when it is not disturbed by bursts and pauses. This value is approximately in the same range for DSS and SRS and lower in case of the UIS that reflects uptake inhibition and diminished DA release. This points towards strong low frequency fluctuations in this scenario, caused by an impaired reinstatement rather than fast oscillations due to transient DA release.

It becomes obvious that, in case of uptake inhibition, the balance between release and uptake is heavily disturbed. In the default setting and also in the SRS tonic DA appears noisy (larger random jumps) but stable (fast reinstatement). In contrast, DA tone in the UIS exhibits smaller random jumps, but is overlaid by longer fluctuations caused by a low reversion rate. Thus, when uptake is inhibited, the overall heightened DA tone together with a reduced capacity to effectively decrease concentration during pauses or after bursts of DA neuronal firing potentially impact on behavior. Additionally, the long fluctuations could easily be misinterpreted as systemic, i.e. as small phasic signals. In turn, small phasic signals might not be detectable due to the relatively high amplitude of the observed fluctuations. In other words, our results suggest a low signal-to-noise-ratio in scenarios of uptake inhibition, which has to be further investigated with simulations of DA bursts and pauses. Finally, not only DA tone but also extra-synaptic DA variability might contribute to altered behavior during uptake inhibition. In fact, in neuroimaging research, brain signal variability has been linked to cognitive performance and was suggested to be considered as a functional property of the human brain [52–54].

#### Spatial variability

In the previous paragraph we discussed temporal characteristics of the volume-averaged extra-synaptic DA concentration. However, throughout the volume there exist strong concentration gradients, which can be very steep near release sites. In default parameter setting, where DA tone is in the range of 10^1^ *nM*, concentration gradients in the range of 10^4^ *nM*/*μm* can occur milliseconds after such release according to our model of a single terminal release event (see *’Synaptic transmission’*). This wide concentration range indicates a very complex spatio-temporal pattern of DA concentration, with very transient occurrence of high values. On a coarser resolution, using fast-scan voltammetry—a method that averages over terminals within a radius of 75*μm* from the electrode—transients of 10 to 100-fold increased DA concentration have been observed [11, 55]. Thus, for our further considerations two aspect will be crucial: First, whether a method measures DA directly or indirectly and second, its temporal and spatial resolution.

**Fig 6:**
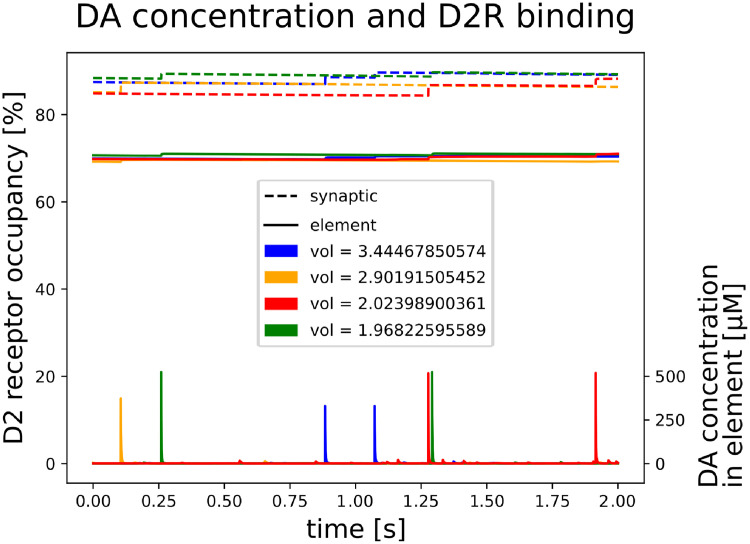
Extra-synaptic DA. DA concentration and D2R binding characteristics for four randomly chosen finite elements containing a synaptic terminal. Depicted at the bottom is DA concentration within the finite element of the tonic model, i.e. extra-synaptic DA concentration within a radius of ~ 0.8*μM* from the terminal. Large concentration peaks indicate DA release from this synapse, while smaller peaks are caused by DA release from neighboring terminals. As the same amount of DA molecules is released every time, peak concentration in larger volumes (coarser resolutions) are smaller. The upper (dotted lines) and middle graphs (solid lines) illustrate slow D2R binding in a short time window for the SRS in synaptic (dotted) and peri-synaptic (solid) space.

In Fig 6 with the bottom graphs we illustrate DA concentration within four randomly chosen finite elements of the tonic model, i.e. extra-synaptic DA concentration within a radius of ∼ 0.8*μM* from the terminal. Large concentration peaks indicate DA release from this synapse, while smaller peaks are caused by DA release from neighboring terminals. As the same amount of DA molecules is released every time, peak concentration in larger volumes (coarser resolutions) are smaller. With our simulations we found that volume averaging strongly simplifies the true dynamics of dopaminergic transmission and can in fact have severe consequences when inferring post-synaptic effects. In particular, if we calculate volume-averaged receptor occupancy from the spatio-temporally varying concentration, the result crucially depends on the order of (a) volume averaging and (b) applying the transform function *F*_*D*2*R*_(*x*):

$$\begin{array}{c} \underset{temporal1}{\underset{︸}{\frac{1}{N}\sum_{e\in \{FE\}}F_{D2R}\left(C\left(e\right)\right)}}\neq \underset{temporal2}{\underset{︸}{F_{D2R}\left(\frac{1}{N}\sum_{e\in \{FE\}}C\left(e\right)\right)}} \end{array}$$

where *C*(*e*) is the concentration in the finite element *e* within the set of all finite elements FE and *N* is the number of finite elements. This order effect is caused by the non-linearity of *F*_*D*2*R*_(*x*), being either the Michaelis-Menten kinetics or dynamic slow or fast receptor binding. As an example, for the model of fast receptor binding, *temporal*1 = 57.1% while *temporal*2 = 67.8%. This might have practical implication when interpreting imaging data that is restricted to immanent resolution and, particularly, when drawing conclusions about DA concentration from PET measurements. Here, binding of tracers and hence binding of competing endogenous DA to D2Rs is frequently used to infer concentration of DA. Specifically, it is important bridge to observations using PET—a method that can be applied to human research—with findings from other methods that can not be used in human studies. In the following we seek evidence for the hypothesis postulated by Laruelle. For that we will rely on *temporal*1 as the more realistic value for tonic D2R occupancy since it should be less corrupted by spatial resolution.

Note however, for our models of dynamic receptor binding we choose on- and off-kinetic parameters such that the resulting dissociation constant is equal to what has been observed on a spacial scale that is coarse compared to the resolution of our model (see ‘Materials and methods’). In this case, tonic DA concentration corresponds to receptor binding given by temporal2. Two aspects have to be considered here: first, the above spacial averaging assumes homogeneous distribution of D2Rs across the extrasynaptic space and second, parameters derived on a macro-scale resolutions (temporal and spatial) may not be equivalent on a micro-scale. Alternatively, parameters for our simulations on micro-scale could be adjusted such that temporal1 equals the empirical observations. However, this goes beyond the scope of this article and is not necessary for the following considerations.

#### Laruelle’s hypothesis

We analys the spatio-temporal DA concentration from the stimulated release (SRS) and uptake inhibition simulation (UIS). The criterion for our simulations, that would support Laruelle’s hypothesis, is an excessive synaptic D2R occupation in SRS compared to UIS. DA tone for default setting simulation (DSS), SRS and UIS leveled at 40.1*nM*, 51.8*nM* and 511.1*nM*, respectively. With our models of dynamic slow and fast D2 receptor binding we obtain the following results.

First we discuss fast binding. For the SRS, on average, DA binds to 57.08% of the D2Rs in the entire volume, assuming those receptors are homogeneously distributed. D2 occupancy is only slighly increased in peri-synaptic space (57.6%) and slighly more inside the synapse (58.2%). Thus a non-homogeneous distribution of D2Rs, i.e. denser near a terminal, can result in an overall D2R occupancy <58.2%. In contrast, for the UIS we obtain comparable estimates across extracellular compartments of approx. 95.3% D2R occupancy. Consequently, with the model of fast receptor binding we can not confirm Laruelle’s hypothesis.

More interesting appears the model of slow binding: For the SRS, on average and under the assumption of homogeneity, DA binds to 57.08% of the D2Rs in the entire volume. Specifically, in peri-synaptic space 70.11% of the D2Rs are being occupied and 87.4% inside the synapse. This difference across different compartments is not pronounced in the UIS. Here our model yields 95.35% in peri- and 95.81% occupancy in synaptic space. High DA concentration in the UIS saturates D2Rs across the entire extracellular volume. Since synaptic and peri-synaptic D2R occupancy in the SRS is much lower compared to the UIS we can not conclude comparable occupancy between these two settings. However, for the SRS, we found differences across compartments. In turn, this means that for PET tracers challenges with endogenous DA can be different in synaptic, peri-synaptic and extra-synaptic space.

In summary, the discrepancy between PET and microdialysis observations can only in part be explained by different challenges across the extra-cellular space, but not completely. This leads to the conclusion that other methodological issues are relevant here. First the observations, i.e the simultaneous PET-microdialysis-measures are from different studies on either nicotine or amphetamine administration and not within the same species. Second, as Laruelle also discussed in his work, even simultaneous intra-subject measures are not always consistent.

## Conclusion

We have proposed a new computational model for DA transmission integrating release from multiple terminals, diffusion and uptake of DA. To our knowledge it is the only model of multiple release sites, to date, that considers both, synaptic and volume transmission. The importance of synaptic transmission has been a matter of debate. With our synaptic model we simulated DA concentration in and near the synaptic cleft. After vesicle release most DA molecules leave the synaptic cleft within less than a millisecond due to fast diffusion. Here peri-synaptic DATs are saturated at early high concentrations.

Experimental observations have led to the conclusion that DATs limit DA spillover [13, 30] and to the discussion about volume transmission versus synaptic transmission. This discussion was based on empirical evidence in midbrain. Here, somatodentritic release mainly serves to regulate the rate and pattern of firing of the DA neurons and hence release into striatum. Synaptic release sites are sparse, they exist in VTA, but not SNc, and their role remains to be determined [33]. For our purpose we simulated DA release in striatum. Here the majority of terminals is non-synaptic [56], which is classically an argument for volume transmission. In addition, our simulations suggest that strongly pronounced DAT activity at the presynaptic terminal can not prevent DA from escaping the synaptic cleft. Directly after DA release, diffusion rather than uptake is the dominating process.

However, uptake seems not only critical for a balanced DA tone, but intensively shapes the extra-synaptic DA signal near release sites. Thus, neither supporting an exclusive intra-synaptic nor a population signal (volume transmission), overall, our simulations show that DA from a terminal, synaptic or non-synaptic, is highly localized to the release site. Hence, it is likely that autocrine signals are mediated, by a cell’s own DA. This does not exclude a pooling effect of DA release from multiple terminals in line with volume transmission.

With respect to post-synaptic effects on neurons in the projection area, we again emphasize that DA is highly localized to its release site, suggesting direct signaling from cell to cell, rather than from cells forming synapses in neighboring distance. In addition, inside synaptic junctions DA concentration gains levels that do not occur with non-synaptic terminals. Beyond the synapse, with models of slow and fast D2R receptor binding kinetics, the difference between synaptically and non-synaptically released DA does not translate into differences in binding. For both release modes the effect on D2R binding was negligible further than half the distance of neighboring synapses with models of slow and fast binding kinetics. Again, this supports the privacy of terminals within their front yard, but does not exclude a mechanism that is based on a population effect. Volume transmission remains an important aspect of DA signaling characterizing the average population activity, hence detecting changes in the amplitude of tonic activity or switches between tonic and burst modes.

However, so far our conclusion about the specificity of synaptic DA was primarily based on the discussion about D2 receptor binding. It is likely that synaptic and also non-synaptic DA is crucial for modulating co-occurring synapses from limbic and cortical afferents. Thus, in addition to the view that the DA system is driven by volume transmission, essential for pre-synaptic control, we suggest to consider the possibility that synaptic transmission is functionally relevant for post-synaptic modulation and hence learning (Fig 1).

Importantly, in his work, Berke [57] reconciles the distinct association between motivation and tonic DA versus learning and phasic DA. In this context, (peri-)synaptic vs. volume transmission might be an alternative candidate mechanism that allows to distinguish between motivational and teaching signals of DA.

Comprising small-scale and large-scale simulations, our model is suitable to investigate hypotheses concerning a single synapse and also a population of cells. As a first application of our large scale model we assessed the spatio-temporal variability of DA concentration in a volume of [50μm]^3^ containing ∼13000 terminals. We showed how temporal concentration variability in extra-synaptic space can be described by an Ornstein-Uhlenbeck process. With this approach we identified differences between healthy DA transmission, enhanced DA release and uptake inhibition. Importantly, from an empirical point of view temporal variability in DA concentration depends on the sampling resolution of the measurement method which in turn can have severe impact on the interpretation of results. With their recent findings Berke and colleagues challenge the view that motivation is mediated by slow changes in tonic dopamine cell firing [57, 58].

The analysis of our simulated DA concentration time series further showed how strongly extra-synaptic DA concentration varies across space and how spatial averaging affects inference on receptor occupancy and subsequent post-synaptic effects. Again, this might have direct implications for the interpretation of neuroimaging measurements such as PET, where competitive binding of radioligands to D2Rs has been inversely related to the availability of endogenous DA.

Physiological measures of DA concentration are either indirect (PET, SPECT, fMRI), invasive (microdialysis, optogenetics) or both and suffer from resolution limitations or poor signal-to-noise-ratio. Computational modeling advances our understanding of dopamine transmission and the complex underlying mechanisms in addition to empirical observations. Furthermore, it can be used to postulate hypotheses before expensive experiments are being conducted or to consolidate apparent discrepancies between results from different empirical observations. Our model may be used to mimic pharmacological manipulations (e.g. the administration of amphetamine or L-DOPA) or DA-related disease and disorders.

In this work, we tested a hypothesis postulated by Laruelle [11]. Based on empirical observations, he proposed that the challenge at D2Rs between endogenous DA and PET tracers is occurring primarily in the synaptic space when DA release is enhanced. In contrast, if DA uptake is inhibited, then this challenge takes place mainly in the extra-synaptic space. This could explain why amphetamine administration (i.e. uptake inhibition) elevates DA concentration levels far beyond elevated levels following nicotine administration as measured with microdialysis, but at the same time reduces D2R binding potential to a comparable amount as measured with PET. We indeed found differences between the synaptic, peri-syaptic and extra-synaptic compartments. Our model suggests the possibility for enhanced challenges in synaptic compared to extra-synaptic space following nicotine administration. However, this could not completely explain the strong difference in concentration occurring in parallel with no difference in D2R binding.

Despite some limitations and discrepancies in empirical research concerning resolution, expense and inference, we want to emphasize the importance of using different empirical methods that have previously established theories for dopamine transmission. These merging theories were necessary to build simulation tools like the one presented here. In turn, model simulations can provide a level of detail not observable with empirical methods or can help to disentangle effects on indirect measures. In that sense, we would like to encourage the combination of empirical and simulation methods, such that they optimize each other. Here we presented such a simulation tool, reported its potentials and limitations and applied it to current research of interest.

## Materials and methods

### Principal processes and model architecture

Our model implements the general process of DA transmission between brain regions, whereby our default parameters account for transmission to striatal regions. The three dominating processes that control DA distribution in extra-cellular space are *release, diffusion* and *uptake*. DA is being released (exocytosis) from randomly distributed synaptic and non-synaptic terminals of neurons that fire in a Poisson-distributed manner. From previous model simulations, Rooney and Wallace [31] propose partial vesicle content release between 0.1 and 21%. In our default model we used full content release with a release probability of 6% per spike as suggested by Dreyer and colleagues [10]. Diffusion strongly depends on the volume fraction of the extracellular space (*α*) and its tortuosity (λ). Both vary across species, brain regions and age [59]. Diffusion can thus be described by the following partial differential equation, where *D* is the free diffusion coefficient and *C*(*x*, *t*) denotes concentration at time *t* and point *x*:

$$\begin{array}{c} \frac{dC(x,t)}{dt}=\tilde{D}*\Delta C\left(x,t\right),\text{where}\;\tilde{D}=\frac{D}{\lambda^{2}}\;. \end{array}$$ (1)

Our model considers isotropic diffusion which is only limited by cellular borders on the synaptic level and by the boundaries of the simulation space. The latter was restricted to zero flux.

$$\begin{array}{c} \frac{dC(x,t)}{dt}=0,\text{for}\;x\in \partial V\;, \end{array}$$ (2)

where ∂*V* is the boundary of the volume. Note that for evaluations of DA transmission we omitted a small strip from the border of the simulation space (by default: 1*μ*m from each side of the [50*μ*m]³ cube), in order to minimize artificial boundary effects.

DA uptake (endocytosis) is realized via DAT. This process can be described by the following differential equation, the Michaelis-Menten (MM) enzyme kinetics

$$\begin{array}{c} \frac{dC(x,t)}{dt}=-\frac{V_{max}*C\left(x,t\right)}{K_{m}+C\left(x,t\right)}\;, \end{array}$$ (3)

where *V*_*max*_ and *K*_*m*_ denote the maximum uptake rate and the dissociation constant respectively.

Our model was implemented using Fipy [60], a python-based finite-volume-method to solve the differential equation integrating diffusion (Eq 1) and uptake (Eq 3).

### Resolution

**Fig 7:**
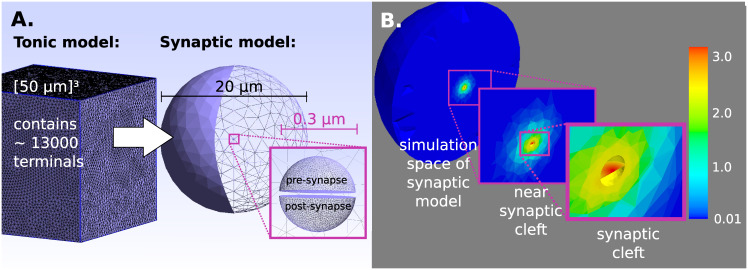
Model. **A)** Tonic (large-scale) and synaptic (small-scale) model. The tonic model contains more than 13000 finite elements that are associated with synaptic and non-synaptic terminals. For non-synaptic terminals the whole vesicle content is released into the respective finite element, while synaptic terminals refer to the synaptic model. **B)** DA diffusion from a synaptic terminal. Concentration 0.1 ms after release.

Our model comprises two levels of resolution, the large-scale tonic and the small-scale synaptic level. Separation into these two levels allows simulations to captures release, diffusion and uptake both in extra-synaptic space and inside synaptic clefts with high computational efficiency. The synaptic model accounts for the synaptic and near-synaptic space. It is implemented on a very narrow mesh in the range of 10^−3^ *μm* and simulations are performed at very high temporal resolution of 10^−5^ *ms*. For each synaptic terminal such a synaptic model is then embedded into the tonic model which encompasses the entire simulation space and is coarser in space (10^0^ *μm*) and time (0.25*ms*) (Fig 7A). Extending the high resolution of the synaptic model to the entire simulation space would result in infeasible computational effort. Thus, in our tonic model, the synaptic model is used for the finite elements where a releasing terminal is located and only as long as additional DA molecules enter the extra-synaptic space. During every iteration of the tonic model this amount of DA molecules from the synaptic model is transferred into the respective finite element of the tonic model.

### Tonic model and volume transmission

In our approach, extra-synaptic DA is simulated within the tonic model. For non-synaptic terminals, the full content of a DA vesicle is released into the respective finite element instantaneously. For synaptic release, on the other hand, a fraction of the vesicle content is gradually released according to the calculations from the finer grained synaptic model as described below. Running the synaptic model with baseline concentrations between 0.001 and 500*nM* revealed that within this range DA spillover does not depend on the baseline concentration and that more than 97% of the released molecules have left the synaptic cleft within the first 0.25*ms*. For both synaptic and non-synaptic terminals, similarly to earlier point source models [8–10], DA then diffuses in all directions isotropically. In accordance with these models we further assume volumetric Michaelis-Menten uptake on the tonic level. Volume transmission is thus described by the following equation, combining diffusion and uptake:

$$\begin{array}{c} \frac{dC(x,t)}{dt}=\tilde{D}*\Delta C\left(x,t\right)-\frac{V_{max}*C\left(x,t\right)}{\alpha (K_{m}+C\left(x,t\right))}\;. \end{array}$$ (4)

This equation calculates concentration in the entire tissue. To obtain concentration in extra-cellular space, the resulting concentration has to be divided by *α*.

Previous simulation studies of DA transmission [8–10] based their calculations on this equation and the assumption of low concentrations *K*_*m*_ >>*C*, which reduces the MM kinetics (Eq 3) to the linear uptake case:

$$\begin{array}{c} \underset{K_{m}>>C}{lim}\frac{dC(x,t)}{dt}=-\frac{V_{max}}{K_{m}}C\left(x,t\right)\;. \end{array}$$

Under this assumption there exists an analytical solution of (Eq 4), which has been demonstrated by Nicholson [24]. Under normal conditions, tonic DA levels of approximately 4 − 50*nM* would satisfy this assumption, but large concentration variability exists within the extra-synaptic volume. High concentration values occur in the vicinity of release sites that would violate the assumption *K*_*m*_ >>*C*. Instead, if *K*_*m*_ <<*C* uptake approximates its maximum, i.e. *V*_*max*_.

### Synaptic model and synaptic transmission

Very high concentrations also appear inside the synaptic cleft after vesicle content release, although these high concentrations are transient. Importantly, with our numerical method of finite elements we can apply our model to any concentration values in the absence of an analytical solution.

DA can only escape the synaptic cleft where it is not constrained by cellular membranes. A point source model may not be appropriate here. Since in striatum approximately 35% of the nerve terminals are synaptic [61] a more realistic model should incorporate this specific geometry. In our model the synapses appear as discs with a diameter of 150*nm* and a distance of 15*nm* between pre- and post-synaptic neuron [23]. DA release occurs centrally from the membrane of the pre-synaptic cell. Fig 7B zooms into the synaptic model towards the synaptic cleft. The disk-like synapse appears in red, as DA has just been released. The ‘empty space’ above and below the disk resemble the pre- and post-synapse.

The diffusion coefficient D* on the synaptic level might differ from volume transmission due the different physical and chemical conditions. However, in the absence of empirical measurements on the synaptic level, we used the same coefficient in both models.

To compare diffusion from non-synaptic terminals to synapses, we also designed a single half shell and released DA from its center, comparable to the synaptic case of two half shells and used identical parameters.

In addition, we wanted to test, if strongly pronounced DAT uptake at the pre-synaptic terminal can prevent DA from escaping the synaptic cleft. In this context, we implemented surface uptake, that is two-dimensional. We derived respective values for the maximum uptake capacity $V_{max}^{sur}$ and the dissociation constant $K_{m}^{sur}$ as follows: For a single axon, we assume a cylinder with an average length *l* = 467000*μm* [62] and diameter *d* = 2 * *r* = 0.25*μm*. The ratio between axonal volume (*V*^*A*^) and axonal surface (*S*^*A*^) is then 1*LU*^3^: 15*LU*^2^ (LU = length unit) and the volume of a single axon is determined by:

$$\begin{array}{c} V^{A}=\pi *r*l=22923.8089\mu m^{3} \end{array}$$

Given that there are on average 370,000 terminals per axon and 0.104 terminals per μm^3^ striatum (see references in [10]), we can conclude that per striatal volume unit there exist approximately 0.00644 axonal volume. Since, in striatum, the extrasynaptic volume fraction has been estimated to be 0.21 [24], the ratio of extra-synaptic volume (*V*^*E*^) to axonal cell volume is approximately 1: 0.03. According to the ratio *V*^*A*^: *S*^*A*^, it follows that 1*LU*^2^ axonal membrane surface should have the same dopamine uptake capacity as approximately 2.174*LU*^3^ extra-synaptic volume and therefore we assume:

$$\begin{array}{c} V_{max}^{sur}\approx 2.174*V_{max}^{vol}\approx 2*4.1\mu M/s \end{array}$$

$$\begin{array}{c} K_{m}^{sur}\approx 2.174*K_{m}^{vol}\approx 2*0.21\mu M \end{array}$$

Above, we have derived 2-dimensional uptake from empirical measures of 3-dimensional parameters. Interestingly, we get similar results if we start from a one-dimensionl perspective. A single DAT is capable of cycling 2-5 DA molecules/s and there appears to be 175-350 (median = 237.5) DATs in 1*μm*^3^ striatal volume [23], which corresponds to 0.0966 *μm*^2^ axonal surface. Here, DATs cycle about 1131 molecules. Then, with *N*_*A*_ being the Avogadro constant, we obtain:

$$\begin{array}{c} V_{max}^{sur}\approx \frac{5molecules}{s}*\frac{1131}{\mu m^{3}}*\frac{mol}{N_{A}}\approx 9.39\mu M/s \end{array}$$ (5)

### Receptor occupancy

Similar to uptake via DAT, receptor occupancy can be described by the Michaelis-Menten kinetics Eq 3. Using *V*_*max*_ = 1 for full occupancy and *EC*50 instead of *K*_*m*_ we get:

$$\begin{array}{c} \text{Occupancy}\left(x,t\right)=\frac{C(x,t)}{EC50+C(x,t)} \end{array}$$

EC50 is the effective concentration in equilibrium with 50% occupancy. Values for D1R and D2R are listed in S1 Table. This is a very simplistic model for the dynamics of receptor occupancy and has been recently advanced in a computational model [16]. This model implements dynamic on- and off kinetics. We solved this equation numerically. Using the Euler-method we obtained the following recursive equation for D2 occupancy (Occ):

$$\begin{array}{c} \text{Occ}\left(x,t+1\right)=\text{Occ}\left(x,t\right)+dt*(D2_{on}*\text{Conc}\left(x,t\right)*\left(1-\text{Occ}\left(x,t\right)\right)-D2_{off}*\text{Occ}\left(x,t\right)) \end{array}$$

Where *D*2_*on*_ and *D*2_*off*_ reflect the on- and off binding rate. For slow and fast binding kinetics these parameters were taken from [16] and [26], respectively.

### Ornstein-Uhlenbeck-Process

The Ornstein-Uhlenbeck Process is a stochastic process used to model mean reverting behavior. *X*_*t*_ is an Ornstein-Uhlenbeck process (OUP) if

$$\begin{array}{c} dX_{t}=\lambda \left(\mu -X_{t}\right)dt+\sigma dW_{t}\;, \end{array}$$

where *μ* is the mean reversion level to which the process tends to revert, λ is the mean reversion rate, *σ* measures the volatility of the process and *W*_*t*_ is a Wiener process. The process has a stationary distribution *X*^*stat*^ that is a normal distribution:

$$\begin{array}{c} X^{stat}∼N\left(\mu ,\sigma^{2}/2\theta \right) \end{array}$$

We used an OUP to describe the course of spatially averaged extra-synaptic concentration, which fluctuates around a mean level, i.e. DA tone, according to the balance between release and uptake. The OUP was fitted in R using the mle() function.

### Limitations

Our model could be extended to some more aspects of the DA system. First, metabolism of DA that would regulate the impact of DA synthesis has not yet been considered. We expect this mechanism to have very little impact as it occurs at a sufficiently slow rate, not affecting transient changes in concentration [63] and our simulations of up to 100 seconds. Second, the model could be advanced by a continuous firing frequency depending on D2 auto-receptor activation. This was for example demonstrated in [64]. Third, in our model, DA molecules bound to receptor sites have not been subtracted from the extra-cellular concentration. This should not affect steady-state analysis, but there is a potential effect during phasic DA release. Fourth, vesicle content should not be released at an instance but over the time of 10^−1^ *s* [48].

## Supporting information

S1 Table Table of model parameters. Table splits into parameters taken from previous computational modeling studies and additional parameters for synaptic specifity.
(PDF)

S2 Table Different Michaelis-Menten uptake parameters reported in the literature. Values differ across species and within a species striatum.
(PDF)

S3 Table Physiological measurements of endogenous DA in humans. Mean values range between 23 and 72*nM*.
(PDF)

S4 Table Ornstein-Uhlenbeck process. Maximum likelihood fits to default simulation (D1-D3) revealed three local maxima depending on initialization and one global maximum for the uptake inhibition simulation (UI).
(PDF)
