# Supplementary material for: Dopamine release, diffusion and uptake: A computational model for synaptic and volume transmission
Source: PLoS Comput Biol. 2020 Nov 30;16(11):e1008410. doi: 10.1371/journal.pcbi.1008410 (PMC7728201; doi:10.1371/journal.pcbi.1008410)
Supplement: S1 Table — Table splits into parameters taken from previous computational modeling studies and additional parameters for synaptic specifity. (PDF) [file pcbi.1008410.s001.pdf]

Supporting information

S1 Table Table of model parameters. Table splits into parameters taken from previous computational modeling studies and additional parameters for synaptic specifity.

parameters taken from [1]	value		value in [2]
Volume fraction	0.21		identical
Eff. diffusion constant	322	$\mu m^2/s$	identical
Terminal density	0.104	μm^{-3}	-
Terminals per axon	370000		-
Release probability	6	%	-
Molecules released	3000		9800
Uptake parameter V_{max}	4.1	$\mu M/s$	identical
Uptake parameter K_m	0.21	μM	identical
EC50-D1R	1.0	μM	identical
EC50-D2R	0.01	μM	identical
Tonic firing frequency	4	Hz	-
**additional parameters**	**value**		**Reference**
Distance pre- to post-synapse	0.015	μm	[3]
Diameter synaptic cleft	0.3	μm	[3]
Proportion non-syn. terminals	0.65		[4]

References

1. Dreyer JK, Herrik KF, Berg RW, Hounsgaard JD. Influence of Phasic and Tonic Dopamine Release on Receptor Activation. Journal of Neuroscience. 2010;30(42):14273–14283. doi:10.1523/JNEUROSCI.1894-10.2010.

2. Rice ME, Cragg SJ. Dopamine spillover after quantal release: Rethinking dopamine transmission in the nigrostriatal pathway. Brain Research Reviews. 2008;58(2):303 – 313. doi:https://doi.org/10.1016/j.brainresrev.2008.02.004.

3. Garris P, Ciolkowski E, Pastore P, Wightman R. Efflux of dopamine from the synaptic cleft in the nucleus accumbens of the rat brain. Journal of Neuroscience. 1994;14(10):6084–6093. doi:10.1523/JNEUROSCI.14-10-06084.1994.

4. Descarries L, Watkins KC, Garcia S, Bosler O, Doucet G. Dual character, asynaptic and synaptic, of the dopamine innervation in adult rat neostriatum: A quantitative autoradiographic and immunocytochemical analysis. Journal of Comparative Neurology. 1996;375(2):167–186. doi:10.1002/(SICI)1096-9861(19961111)375:2¡167::AID-CNE1¿3.0.CO;2-0.
