# Supplementary material for: Dopamine release, diffusion and uptake: A computational model for synaptic and volume transmission
Source: PLoS Comput Biol. 2020 Nov 30;16(11):e1008410. doi: 10.1371/journal.pcbi.1008410 (PMC7728201; doi:10.1371/journal.pcbi.1008410)
Supplement: S2 Table — Values differ across species and within a species striatum. (PDF) [file pcbi.1008410.s002.pdf]

Supporting information

S2 Table Different Michaelis-Menten uptake parameters reported in the literature.

Source	Animal	V_{max} $[\mu M/s]$	K_m $[\mu M]$
[1]	rat	2.78	0.16
[2]	guinea-pig	2.68	0.21
[2]	marmoset	3.27 (ventromedial) - 5.02 (dorsolateral)	0.21
[3]	rat	3.20	0.11 (medial) - 0.15 (lateral)
[3]	6-OHDA lesioned rat	depended on degree of lesion	0.21 (medial) - 0.02 (lateral)

References

1. Kawagoe KT, Garris PA, Wiedemann DJ, Wightman RM. Regulation of transient dopamine concentration gradients in the microenvironment surrounding nerve terminals in the rat striatum. Neuroscience. 1992;51(1):55 – 64. doi:https://doi.org/10.1016/0306-4522(92)90470-M.

2. Cragg SJ, Hille CJ, Greenfield SA. Dopamine Release and Uptake Dynamics within Nonhuman Primate Striatum In Vitro. Journal of Neuroscience. 2000;20(21):8209–8217. doi:10.1523/JNEUROSCI.20-21-08209.2000.

3. Bergstrom BP, Garris PA. 'Passive stabilization' of striatal extracellular dopamine across the lesion spectrum encompassing the presymptomatic phase of Parkinson's disease: a voltammetric study in the 6-OHDA-lesioned rat. Journal of Neurochemistry;87(5):1224–1236. doi:10.1046/j.1471-4159.2003.02104.x.
