# Supplementary material for: Dopamine release, diffusion and uptake: A computational model for synaptic and volume transmission
Source: PLoS Comput Biol. 2020 Nov 30;16(11):e1008410. doi: 10.1371/journal.pcbi.1008410 (PMC7728201; doi:10.1371/journal.pcbi.1008410)
Supplement: S3 Table — Mean values range between 23 and 72nM. (PDF) [file pcbi.1008410.s003.pdf]

Supporting information

S3 Table Physiological measurements of endogenous DA in humans.

Source	Tonic level	partial depletion	tracer	method
[1]	72 ± 40 nM $^{*)}$	70%	[^{123}I]IBMZ	SPECT
[2]	23 ± 18 nM $^{*)}$	70%	[^{123}I]IBMZ	SPECT
[3]	27 ± 6 nM $^{**)}$	not reported	[^{11}C]raclopride	PET

References

1. Laruelle Marc, D'Souza Cyril D, Baldwin Ronald M, Abi-Dargham Anissa, Kanes Stephen J, Fingado Christine L, et al. Imaging D2 Receptor Occupancy by Endogenous Dopamine in Humans. Neuropsychopharmacology. 1997;17:162. doi:https://doi.org/10.1016/S0893-133X(97)00043-2 10.1016/S0893-133X(97)00043-2.

2. Abi-Dargham A, Rodenhiser J, Printz D, Zea-Ponce Y, Gil R, Kegeles LS, et al. Increased baseline occupancy of D2 receptors by dopamine in schizophrenia. Proceedings of the National Academy of Sciences. 2000;97(14):8104–8109. doi:10.1073/pnas.97.14.8104.

3. Verhoeff NPLG, Kapur S, Hussey D, Lee M, Christensen B, C Psych , et al. A Simple Method to Measure Baseline Occupancy of Neostriatal Dopamine D2 Receptors by Dopamine In Vivo in Healthy Subjects. Neuropsychopharmacology. 2001;25:213. doi:https://doi.org/10.1016/S0893-133X(01)00231-7 10.1016/S0893-133X(01)00231-7.
