# Supplementary material for: Dopamine release, diffusion and uptake: A computational model for synaptic and volume transmission
Source: PLoS Comput Biol. 2020 Nov 30;16(11):e1008410. doi: 10.1371/journal.pcbi.1008410 (PMC7728201; doi:10.1371/journal.pcbi.1008410)
Supplement: S4 Table — Maximum likelihood fits to default simulation (D1-D3) revealed three local maxima depending on initialization and one global maximum for the uptake inhibition simulation (UI). (PDF) [file pcbi.1008410.s004.pdf]

Supporting information

S4 Table Ornstein-Uhlenbeck process. Maximum likelihood fits to default setting simulation (D1-D9), stimulated release simulation (R1-R9) and uptake inhibition simulation (U1-U9) revealed different local maxima depending on initialization. The label number refers to the set of initial values $(\theta_1, \theta_2, \theta_3)$, which are also displayed in the lower table. The rate and volatility parameter of the default simulation were ~ 35.22 and ~ 0.051, respectively, for all initializations. Similarly, across all initializations the volatility parameter of the stimulated release simulation (SRS) was estimated to be 0.058 The most likely values for the rate parameter of the SRS was 62.75 that resulted from six out of the nine initializations. From the three remaining initialization we obtained two more local maxima that were slightly less likely. Importantly, te ratio $volatility^2/(2 * rate)$ of SRS, the resulting variance of the fitted OUPs, and the variance of the time series matched well for the most likely estimate. For this reason, their higher log-likelihood and frequent occurrence, we adhere to this estimate. For the uptake inhibition simulation (UIS) we found two local maxima, each in four out of the nine initializations. With a log-likelihood of 943484.9 and 941197.3 we estimated the rate parameter to equal 1.675 and 9.569, respectively. The volatility parameter was estimated to be 0.0059 and 0.0061, respectively. The variance of the OUP for the first, more likely fit was closer to the variance of the time series compared to the second, slightly less likely fit. The third local maximum appeared only once in our nine runs of the fitting procedure with a low log-likelihood of 876358.2 and is hence not relevant here. For both likely estimates of the UIS the rate and volatility parameter were lower compared to default and SRS. Thus both estimates are in line with our discussion.

	Level (μ)	Rate (λ)	Volatility (σ)	log-L (σ)	Var ($\frac{\sigma^2}{2\lambda}$)
	**Default setting simulation**				
**D1**	0.03965371	35.2111753	0.05121193	570427.4	$3.72 * 10^{-5}$
**D2**	0.03965394	35.2183883	0.05122465	570427.4	$3.73 * 10^{-5}$
**D3**	0.03965702	35.2183883	0.05122133	570427.4	$3.72 * 10^{-5}$
**D4**	0.03965352	35.2229606	0.05122198	570427.4	$3.72 * 10^{-5}$
**D5**	0.03965592	35.2192394	0.05122418	570427.4	$3.73 * 10^{-5}$
**D6**	0.03965865	35.2132316	0.05122001	570427.4	$3.73 * 10^{-5}$
**D7**	0.03965382	35.2183719	0.05122465	570427.4	$3.73 * 10^{-5}$
**D8**	0.03965436	35.2143727	0.05122309	570427.4	$3.73 * 10^{-5}$
**D9**	0.03965389	35.2183657	0.05122481	570427.4	$3.73 * 10^{-5}$
	**Stimulated release simulation**				
**R1**	0.05215297	62.7324701	0.05767703	670681.0	$2.65 * 10^{-5}$
**R2**	0.05214729	62.7855928	0.05766490	670681.0	$2.65 * 10^{-5}$
**R4**	0.05215302	62.7321789	0.05767695	670681.0	$2.65 * 10^{-5}$
**R5**	0.05215297	62.7318579	0.05767696	670681.0	$2.65 * 10^{-5}$
**R6**	0.05215422	62.7524664	0.05767622	670681.0	$2.65 * 10^{-5}$
**R9**	0.05215206	62.7361885	0.05767656	670218.3	$2.65 * 10^{-5}$
**R3**	0.05223935	100.6784477	0.05816766	669792.7	$1.68 * 10^{-5}$
**R7**	0.05153221	10.6286599	0.05772477	669769.9	$1.57 * 10^{-4}$
**R8**	0.05093523	9.96018024	0.05773519	570427.4	$1.67 * 10^{-4}$
	**Uptake inhibition simulation**				
**U5**	0.5091301	1.355079610	0.00589982	943518.2	$1.28 * 10^{-5}$
**U4**	0.5098724	1.694340840	0.00589602	943498.3	$1.03 * 10^{-5}$
**U6**	0.5129455	1.615480556	0.00597493	943464.8	$1.10 * 10^{-5}$
**U1**	0.5091163	2.033705129	0.00595878	943458.3	$8.73 * 10^{-6}$
**U7**	0.5091840	9.529411347	0.00605851	941239.9	$1.93 * 10^{-6}$
**U8**	0.5086668	9.525544424	0.00614326	941201.9	$1.98 * 10^{-6}$
**U9**	0.5092737	9.607655309	0.00608169	941188.6	$1.92 * 10^{-6}$
**U2**	0.5086786	9.612307512	0.00614556	941158.7	$1.96 * 10^{-6}$
**U3**	0.5086200	66.72938939	0.01046446	876358.2	$8.21 * 10^{-7}$

	**initializations**		
**1**	θ_1	θ_2	θ_3
**1**	1	1	1
**2**	1	10	1
**3**	1	100	1
**4**	0.1	1	1
**5**	0.1	1	0.1
**6**	1	1	0.1
**7**	0.1	10	1
**8**	0.1	10	0.1
**9**	1	10	0.1
